# Tiny Bites, a digital health intervention delivered in early childhood education and care centres to support educators and caregivers to prevent childhood obesity: study protocol for a cluster randomised controlled trial

**DOI:** 10.1136/bmjopen-2025-106436

**Published:** 2025-11-23

**Authors:** Sze Lin Yoong, Melanie Lum, Gloria K W Leung, Nicole Pearson, Helen Truby, Clare Dix, Najma A Moumin, Luke Wolfenden, Jaithri Ananthapavan, Alice Grady, John Wiggers, Tessa Delaney, Lucie Rychetnik, Maria Romiti, Hannah Lamont, Sonya Stanley, Michelle Lim, Chris Oldmeadow, Nadia Mastersson, Rachel Sutherland

**Affiliations:** 1 Global Centre for Preventive Health and Nutrition, Institute for Health Transformation, School of Health and Social Development, Deakin University, Geelong, Victoria, Australia; 2 College of Medicine and Wellbeing, University of Newcastle, Callaghan, New South Wales, Australia; 3 Global Centre for Preventive Health and Nutrition, Institute for Health Transformation, School of Health and Social Development, Deakin University, Melbourne, Victoria, Australia; 4 Department of Population Health, Hunter New England Local Health District, Wallsend, New South Wales, Australia; 5 The University of Queensland, Saint Lucia, Queensland, Australia; 6 University of the Sunshine Coast, Sippy Downs, Queensland, Australia; 7 Institute of Health Transformation, Deakin University, Burwood, Victoria, Australia; 8 Department of Economics, Cost-effectiveness, Deakin University, Burwood, Victoria, Australia; 9 University of Newcastle, Newcastle, New South Wales, Australia; 10 The University of Newcastle School of Medicine and Public Health, Callaghan, New South Wales, Australia; 11 NSW Centre for Population Health, Sydney, New South Wales, Australia; 12 The University of Sydney, Sydney, New South Wales, Australia; 13 Deakin University, Geelong, Victoria, Australia; 14 Hunter Medical Research Institute, Newcastle, New South Wales, Australia; 15 The Prevention Centre, Sax Institute, Sydney, New South Wales, Australia

**Keywords:** Body Mass Index, Health Education, NUTRITION & DIETETICS, Community child health, Randomized Controlled Trial

## Abstract

**Introduction:**

Infant feeding practices in the first 2 years of life are linked to long-term weight trajectories. Despite the importance of obesity prevention interventions, there are no randomised controlled trials (RCTs) evaluating early childhood education and care (ECEC) and primary caregiver-targeted interventions on child weight and feeding outcomes.

**Aim:**

To assess the efficacy of an 18-month digital health intervention (Tiny Bites) delivered to ECEC services and primary caregivers of children aged 4 to ≤12 months on child age-adjusted and sex-adjusted body mass index-for-age z-score (zBMI) relative to usual care control in the Hunter New England (HNE) region of New South Wales, Australia.

**Methods and analysis:**

This type 1 hybrid cluster RCT will include up to 60 ECEC services and 540 children/caregiver dyads. The intervention supports ECEC services and caregivers to deliver recommended responsive feeding practices to infants. ECEC services will receive access to an online assessment platform, training and resources, and implementation support. Primary caregivers will receive text messages, monthly e-newsletters, online links and direct communication from ECEC services. We will assess the impact on child zBMI at 18-month follow-up. Secondary outcomes include duration of consuming any breastmilk, child diet and caregiver responsive feeding practices. We will also assess ECEC policy and practice implementation related to targeted feeding practices, programme cost effectiveness, adverse effects and engagement with the programme (ECECs and caregivers). For the primary outcome, between-group differences will be assessed for paired data using two-level hierarchical linear regression models.

**Ethics and dissemination:**

Ethics approval has been provided by HNE Human Research Ethics Committee (HREC) (2023/ETH01158), Deakin University (2024-202) and University of Newcastle HREC (R-2024-0039). Trial results will be submitted for publication in peer-reviewed journals, presented at scientific conferences locally and internationally and to relevant practice stakeholders.

**Trial registration number:**

ACTRN12624000576527.

**STRENGTHS AND LIMITATIONS OF THIS STUDY**

- The study is a cluster randomised trial, with objective outcomes and prospective trial registration.
- The programme was developed through extensive consultation and informed by behavioural frameworks.
- The sampling frame consists primarily of early childhood education and care services in rural, regional and low socioeconomic status areas.
- The use of self-report assessment for secondary outcomes may be subject to reporting and attrition bias.

## Introduction

 Childhood obesity is one of the most significant public health issues globally, with over 37 million children aged under 5 years estimated to be overweight or obese (in 2022).^1^ In Australia, the most recent population data indicate that approximately 20% of children aged 2–4 years are overweight or obese, with rates being disproportionately higher among those living in rural and socially disadvantaged areas.^2^ The first 2 years of life are characterised by a period of rapid growth and development and lay the foundation for lifelong dietary patterns. Studies show that excessive weight gain in this time is associated with longer-term risk for metabolic issues and clinical obesity.^3 4^ Infant feeding practices in the first 2 years of life are strongly associated with children’s weight gain trajectories and influence developing infants’ taste and dietary preferences,^5^,^7^ the impacts of which have been shown to track into childhood, adolescence and adulthood.^8^,^11^ Therefore, obesity prevention interventions to improve infant nutrition are essential for establishing healthy eating patterns and healthy weight trajectories in the long term.^8 12^

Infant feeding practices include the types and quantities of foods offered, the time and settings in which foods are offered, and primary caregivers’ feeding styles during mealtimes in the first 12 months of life.^13^ To help guide caregivers, Australia’s National Health and Medical Research Council (NHMRC) and the Australasian Society of Clinical Immunology and Allergy have published infant feeding guidelines (in 2012)^14^ and allergy prevention guidelines (in 2020)^15^ that include evidence-based recommendations for infant feeding. For infants <12 months, these include guidance around breastfeeding duration, and when and how to introduce solid foods, including allergy-causing foods.^14 15^ For toddlers 12–24 months, guidance includes recommendations for dietary patterns including no discretionary foods (ie, cakes, pastries, processed meat and sugar-sweetened beverages).^14 16^ Due to their high nutrient needs,^17^ there is no allowance for these foods in the diets of children under 2 years, as these foods are higher in saturated fat, added sugars and salt^16^ and may displace core food consumption.^18^,^20^ From 2 years onwards, the dietary guidelines provide specific recommendations for servings of core foods, including an allowance for discretionary foods, for each population group to meet nutritional needs.^16^

However, research suggests that implementation of infant feeding recommendations during this critical period is inconsistent.^18 21^ Encouragingly, the 2021 Australian Feeding Infants and Toddlers Study reported high breastfeeding rates with 98% of caregivers initiating breastfeeding from birth and over 40% continuing to breastfeed into the second year of life.^22^ In contrast, consumption of some core food groups was low, with two-thirds of toddlers aged 12–24 months not consuming the recommended daily serves for vegetables, and meats and alternatives.^18^ Moreover, 90% of toddlers consumed discretionary foods (sweet and savoury snacks) accounting for >10% of daily energy intake.^18^ A New Zealand study with 625 infants aged 7–10 months reported similar findings, where only 30%–60% of infants consumed vegetables, fruit and meat/protein-rich foods daily.^21^

In addition, emerging evidence^23 24^ encourages caregivers to practise ‘responsive feeding’; a feeding style that may prevent overfeeding by identifying and responding to child hunger and satiety cues, not offering food to comfort or reward, distraction-free mealtimes and caregivers role modelling healthy eating behaviours.^25^ However, studies internationally suggest that caregivers face challenges with implementing responsive feeding techniques due to limited skills and knowledge of recognising appetite cues, lack of access to educational resources and inconsistency of information, and social and cultural norms.^13^ Early Childhood Education and Care (ECEC) services are a key setting to support child nutrition as they reach a large number of young children and their primary caregivers during a foundational period of children’s development.^26^ In 2017, approximately 60% of Australian caregivers returned to work or study when their child was less than 7 months old, with the majority accessing long day care centres.^27 28^ This coincides with a period of significant change in terms of infant developmental milestones and feeding requirements.^14 29^ A study with 13 ECEC services capturing 120 mealtime occurrences in Queensland, Australia found that only 22% of ECEC educators demonstrated enthusiastic role modelling and only 3% consumed fruits and vegetables during mealtimes with children.^30^ ECEC educators report a number of barriers in supporting infant feeding in care, including the need for high levels of professional and technical knowledge, perceived tension between educators, parents and the regulatory system, and a lack of structural and operational support to implement recommended practices.^31^

ECEC-based nutrition interventions for infants that involve caregivers provide a unique and timely opportunity to support both educators and primary caregivers in establishing evidence-based infant feeding practices in the early years to optimise healthy weight trajectories. However, our Cochrane systematic review of 52 ECEC-based nutrition interventions found no randomised controlled trials (RCTs) assessing the impact of interventions on infants’ weight (aged less than 2 years old), and only one RCT assessing vegetable intake in children aged 1–2 years.^32^ Another review summarising the impact of parent-targeted interventions only found one RCT demonstrating a small effect on body mass index-for-age z-score (zBMI); however, the effect was not sustained at scale up.^33^ To address these gaps, we designed the ‘Tiny Bites’ programme to support ECECs and caregivers in promoting healthy infant weight gain trajectories by improving adherence to NHMRC infant feeding guidelines^14^ and evidence-based responsive feeding practices.^25^ To enhance scalability, ‘Tiny Bites’ will employ primarily digital modalities (text messages and web platforms) based on evidence of efficacy for targeting preventive health behaviours, and acceptability among end-users.^34^,^36^

### Objectives

This cluster RCT aims to assess the efficacy of ‘Tiny Bites’, an 18-month digital health intervention delivered to ECEC educators and primary caregivers of children aged 4 to ≤12 months, relative to a usual care control group on child zBMI scores (primary outcome). We also sought to assess the impact of the ‘Tiny Bites’ programme at 18-month follow-up on:

1. Duration child receives any breastmilk.
2. Child consumption of fruits and vegetables, ‘discretionary’ foods and consumption of any sugar sweetened beverages.
3. Primary caregivers’ implementation of recommended infant feeding practices (as outlined in the Australian Infant Feeding Guidelines) and evidence-based responsive feeding behaviours.
4. ECEC implementation of comprehensive nutrition and breastfeeding policies and adoption of evidence-based feeding recommendations including responsive feeding.
5. Costs associated with intervention development, delivery and implementation.
6. Cost-effectiveness of the intervention, expressed as the additional cost per 0.1 zBMI reduction.
7. Potential adverse effects of the intervention (eg, food wastage, caregiver/ECEC staff complaints).
8. Process evaluation measures including intervention engagement and acceptability (as reported by ECEC services and caregivers), and feasibility and fidelity (from the perspective of ECEC services).

### Hypothesis

Relative to a usual care control group, infants aged 4 to ≤12 months at enrolment who receive the Tiny Bites intervention will have a lower zBMI of 0.26 kg/m^2^ at approximately 18 months follow-up.

## Methods and analysis

### Study design and setting

This is a type 1 hybrid cluster RCT conducted with ECEC services and caregiver-child dyads (caregivers and their infants 4 to ≤12 months) located in the Hunter New England (HNE) region of New South Wales (NSW), Australia. This design enables the assessment of the feasibility of the intervention and the potential effects of an implementation strategy on ECEC adherence to healthy feeding practices, while assessing the effectiveness of the intervention in improving child dietary intake of fruit and vegetables. The primary outcome will be between-group differences in the mean zBMI, with data collected at baseline (July to September 2024), and following the delivery of the 18-month intervention (approximately February to April 2026).

This protocol (Version 5.0, 22 October 2024) adheres to the Standard Protocol Items: Recommendations for Interventional Trials guidelines for protocol of clinical trials.

### Participants and recruitment

#### Sampling frame

The sampling frame will consist of ECEC services located within the HNE Local Health District (HNELHD) that caters for children in the targeted age range (ie, Long Day Care centres). Services were removed from the sampling frame if they were located in postcodes where health service programmes have been rolled out in the past 6 months.^37^ Thus, the resulting sample consists of 111 services, of which over 70% are in areas classed as inner regional (using Accessibility/Remoteness Index for Australia classification)^38^ and over 60% are classified as low socioeconomic status (SES) (according to the Socio-Economic Indexes for Areas (SEIFA) classification).^39^ Long daycare centres in Australia typically provide foods to children on menus, with a small proportion requiring parents to pack food from home.

#### Study population

ECEC services and primary caregivers and their infants aged 4 to ≤12 months at the time of recruitment and baseline data collection.

### Eligibility criteria

#### Inclusion criteria

For ECEC services to be eligible, they must: (1) have at least five children aged 4 to ≤12 months enrolled; (2) have internet access and (3) have not participated in an obesity-prevention trial in the past 2 years.

Caregivers will be eligible to participate if: (1) they have a mobile phone with web capabilities (eg, 4G/5G) and (2) they can understand English sufficiently to engage with the intervention, as all intervention resources are only available in English.

Children will be eligible if they: (1) are aged 4 to ≤12 months (inclusive) at recruitment, (2) are present at the ECEC service on recruitment day(s) and (3) have caregiver assent. Families with more than one child of the same age (ie twins, triplets) will be included if they meet the eligibility criteria; however, caregivers will only receive the intervention once.

#### Exclusion criteria

ECEC services will be excluded if they report catering exclusively to children with special needs or are a Department of Education community-run preschool or kindergarten (as these organisations are not covered within the existing ethics arrangement and are unlikely to cater to infants). Children with severe dietary restrictions that limit their ability to consume foods in line with the NHMRC Infant Feeding Guidelines will also be excluded.

### Recruitment procedures

**Figure 1:**
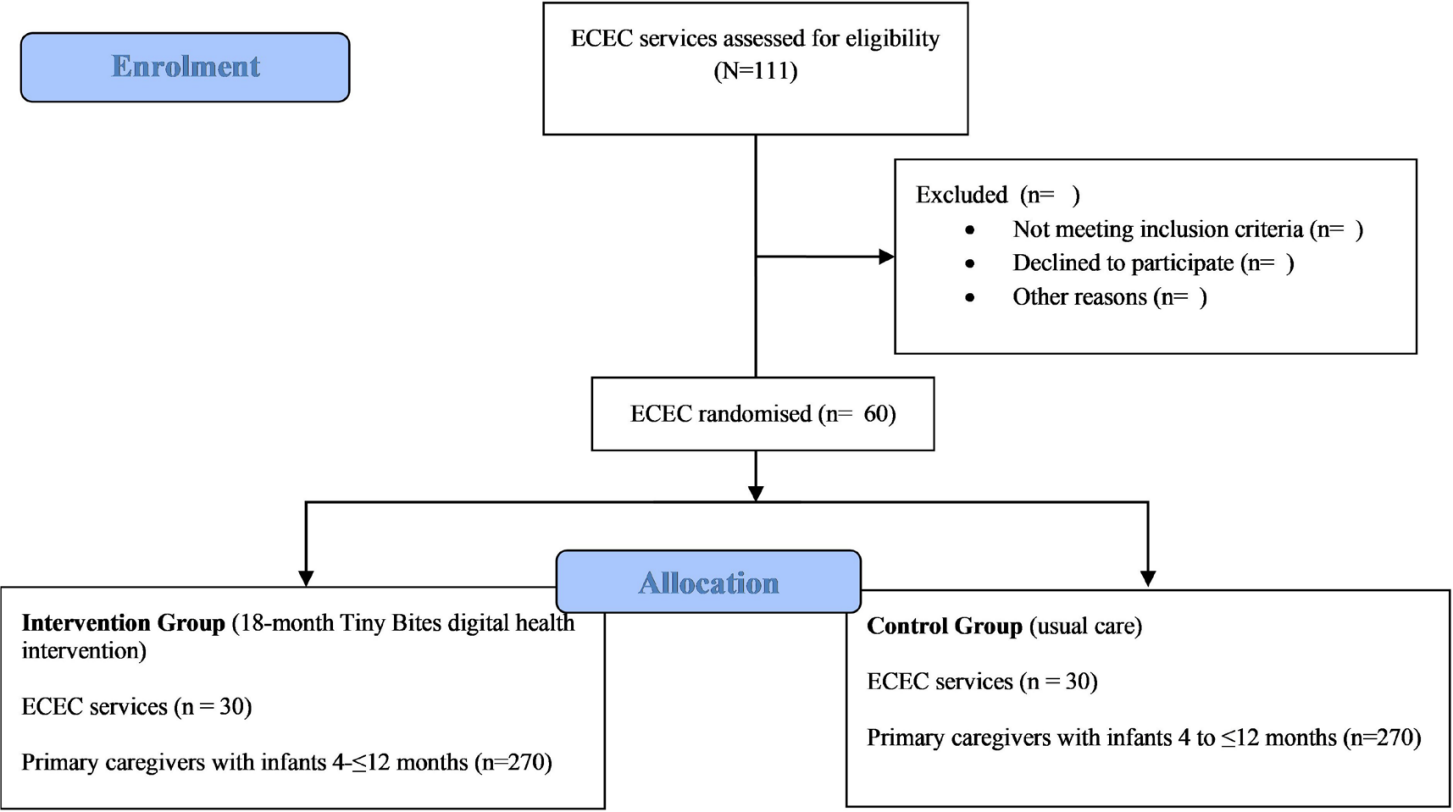
Participant flow through study. ECEC, early childhood education and care.

All ECEC services in the sampling frame will be emailed information statements and consent forms outlining study requirements (see online supplemental file 1). The recruitment process will be overseen by a dedicated recruitment manager. Approximately a week after sending this email, the ECEC services will be contacted by the research team to determine eligibility. If eligible, they will be invited to participate. The ECEC service manager will complete an electronic consent form to participate in the study. For consenting services, the team will attend the service (ie, site visit) ideally on day(s) with the highest infant attendance, to recruit caregivers and commence baseline data collection (see figure 1). Prior to this visit, services will be asked to distribute hard copies of information statements and consent forms (see online supplemental file 2) to caregivers of eligible children and share study recruitment information via their usual forms of communication with caregivers (eg, emails, apps, social media, newsletters).

### Data collection

All trial data will be collected and managed using REDCap, a secure web-based platform, which allows for a complete audit trail of all participant data.^40 41^ On consent, ECEC managers will be asked to nominate an educator working in the infant room (ie, the room that typically cares for children aged 0–2 years). At baseline and follow-up, both ECEC managers and the nominated educators will be invited to complete an online survey. We will also follow up on completion of the surveys at the ECEC service during site visits and/or via phone call by the research team, if required. Child weight and height will be measured during the site visit following caregiver consent. Caregivers will also be invited via email to complete an online survey at baseline and follow-up. A reminder email will be sent 3 days following the initial email. Where surveys have not been completed online within 1 week of the initial email, a research team member will call the caregivers via telephone to prompt completion online or complete over the phone. See table 1 for the complete schedule of enrolment, intervention and assessments.

**Table 1:** Schedule of enrolment, interventions and assessments

	Enrolment		Allocation	Intervention				follow-up	
Time point	*−*t_1_		0	t_1_				t_2_	
Enrolment	July–September 2024		July–September 2024	August 2024–April 2026				April–June 2026	
	Telephone /email	At ECEC service	Email						
Eligibility screen (ECEC services)	X								
Informed consent (ECEC services)	X								
ECEC service visit		X							
Eligibility screen (primary caregivers)		X							
Informed consent (primary caregivers)		X							
Child anthropometric and demographic data collection (primary caregivers)		X							
Allocation (ECEC service+primary caregivers)			X						
Intervention:				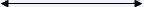					
Assessments:									
	Online	At ECEC service						Online	At ECEC service
Child anthropometrics		X							X
Caregiver and child demographic data	X								
Caregiver feeding practices	X							X	
Breastmilk duration	X							X	
Child fruit and vegetable intake	X							X	
Child discretionary food intake	X							X	
ECEC service characteristics/demographics	X								
ECEC service nutrition and breastfeeding policies	X							X	
ECEC service educator infant and responsive feeding practices	X							X	
Intervention cost (ECEC services+primary caregivers)	X			X	X	X	X	X	
Adverse effects (ECEC services+primary caregivers)	X							X	
Intervention engagement (ECEC services+primary caregivers)								X	
Intervention acceptability (ECEC services+primary caregivers)								X	
Intervention feasibility (ECEC services)								X	
Intervention fidelity (ECEC services)								X	
Relationship Quality (ECEC services+primary caregivers)	X							X	
Self-efficacy (ECEC services+primary caregivers	X							X	
ECEC service communication with caregivers	X							X	

ECEC, early childhood education and care.

### Randomisation

Following collection of primary outcome data (child weight and length), ECEC services will be randomly assigned to the intervention or a usual care control group in a 1:1 ratio. Randomisation will be stratified by Aboriginal and Torres Strait Islander child enrolments (≥10%), to ensure equity of access and SES (dichotomised as high/low SES according to the SEIFA classification^39^ of the ECEC service postcode), given the association between SES and child zBMI.^42^ We will apply block randomisation (4 or 6) to ensure group allocation is approximately equal. Randomisation will be performed using R (V.4.3.2) and completed by an independent statistician following baseline data collection.

### Blinding

ECEC services and caregivers will not be blinded to group allocation. Where possible, data collectors for the primary outcome data will be blinded; however, due to the nature of the intervention, it is possible that some elements of the intervention (ie, posters displayed at the service) may be visible to data collectors at follow-up. The statistician will be blinded to group allocation.

### Intervention design

**Figure 2:**
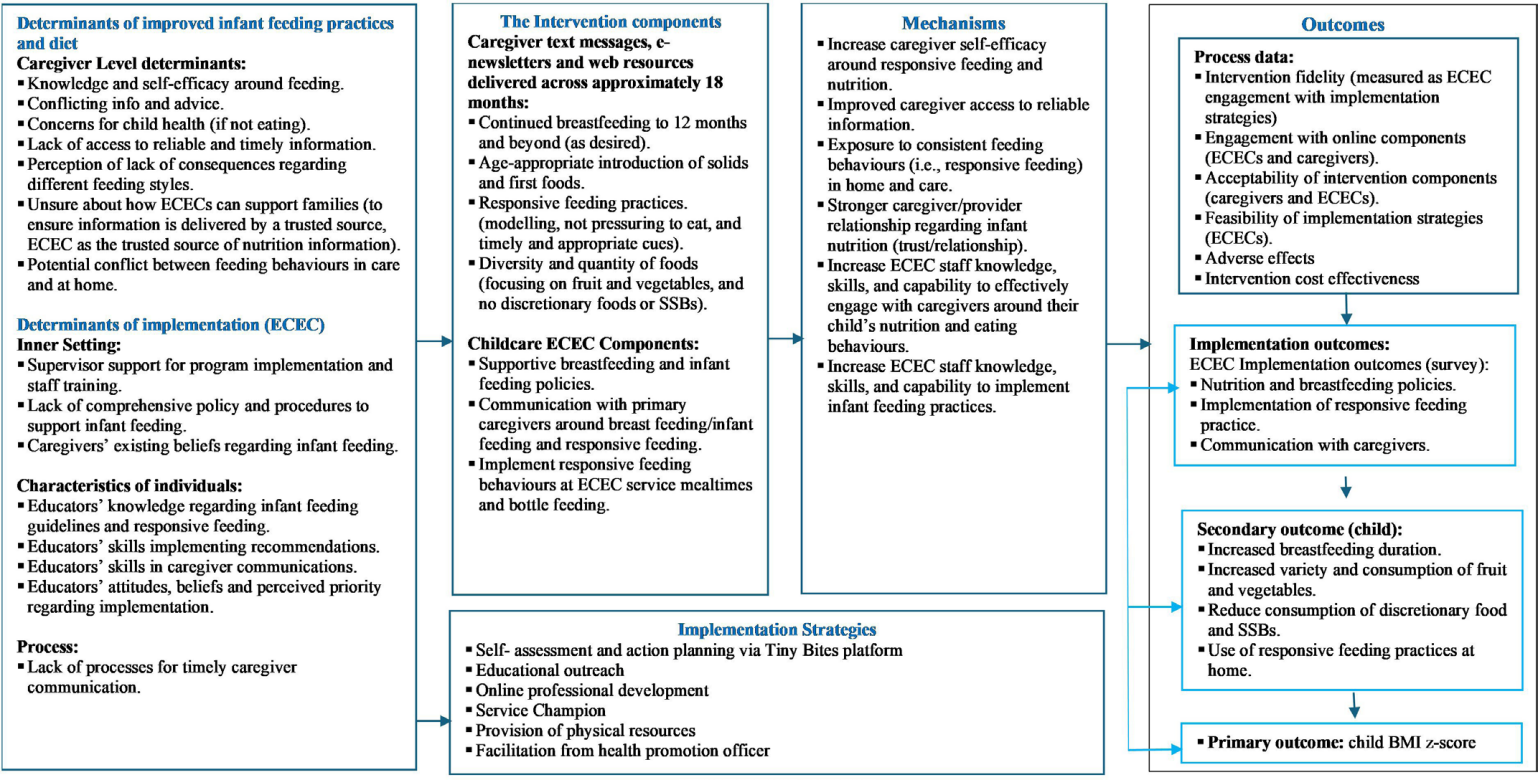
Programme logic model for a type 1 hybrid cluster RCT—the Tiny Bites Programme (60 clusters; 540 caregivers). BMI, body mass index; ECEC, early childhood education and care; RCT, randomised controlled trial; SSB, sugar-sweetened beverages.

We used the Implementation Research Logic Model to outline the intervention processes (see figure 2). This logic model describes the specific practice targets for primary caregivers and ECEC services, the strategies employed to target these practices, how the intervention aims to have an impact (mechanisms) and the intervention and implementation outcomes assessed as part of this process (figure 2).^43^ The specific intervention targets (extending breastfeeding duration, infant feeding and responsive feeding practices, and child dietary intake) were selected as longitudinal evidence reports strong associations between these behaviours and child weight trajectories.^44^,^46^ For ECECs, these targets also align with the broader regulatory frameworks and context for health promotion within the state (NSW) and nationally.^47^,^50^

The COM-B model (capability, opportunity, motivation, behaviour)^51^ was used to inform the development of the intervention. First, we reviewed barriers and facilitators for both ECEC services and primary caregivers when implementing general infant feeding and responsive feeding recommendations via a literature review.^13 52^ Additionally, focus group interviews with 17 ECEC educators and 19 primary caregivers within the HNE region were conducted to explore any context-specific barriers and facilitators to supporting infant feeding and nutrition (see figure 2 for identified determinants). This informed the preferences for specific digital modalities suitable for both ECECs and primary caregivers. The delivey of the caregiver component was informed by the Healthy Beginnings for Hunter New England Kids (HB4HNE) programme,^37 53^ a digital programme targeting caregivers with infants 0–6 months in the HNELHD.^34^ Adaptations were made to address the specific infant feeding and responsive feeding barriers identified through our pilot work.

The identified barriers and facilitators were mapped against the COM-B and used to inform selection of relevant intervention functions and behaviour change techniques (BCTs) to address the specific determinants for ECEC service managers and primary caregivers (see online supplemental file 3 for BCT mapping). This resulted in a multicomponent intervention delivered to ECEC services via an online platform with health promotion officer implementation support and primary caregivers of infants aged 4 to ≤12 months via mobile text messages, e-newsletters and web resources.

### Study treatments

#### Intervention: ECEC services

To support implementation of the targeted practices outlined in figure 2, we will employ the following strategies facilitated by a health promotion officer located within HNELHD. This will be delivered to a nominated educator within the infant room and the service manager. ECECs will:

- Complete the web-based Tiny Bites self-assessment to develop an action plan for implementation. The self-assessment questions relate to having comprehensive policies for breastfeeding, infant feeding and responsive feeding, providing regular and accurate communication as well as resources to primary caregivers around infant feeding and responsive feeding and supporting educators with implementing best practice responsive feeding practices.
- Attend an initial educational outreach visit by the health promotion officer and receive three to four support calls throughout the intervention (facilitation) to support self-assessment, action planning and address any barriers to implementation.
- Appoint a Nutrition Champion for the infant room who will act as the point of contact for the Tiny Bites programme at the service driving implementation, guiding staff, modelling responsive feeding practices and engaging families.
- Receive educational resources to support policy and practice change (eg, policy and orientation templates, hard copy resources, checklists and posters) as well as resources to support regular parent communication.
- Receive access to online professional development including six 15 min self-paced webinars covering infant feeding and responsive feeding practices; and one 35 min self-paced webinar covering nutrition conversations with caregivers.

#### Intervention: primary caregivers

Primary caregivers in the intervention arm will receive:

- Regular text messages (up to 74 over an 18-month period) tailored to their child’s age (from age 4 to 36 months, depending on age at enrolment).
- Monthly e-newsletters via email summarising resources and SMS message content (these newsletters will also be delivered to nominated individuals who may be involved in feeding children, such as grandparents/extended family members). Some SMS messages and digital newsletters will include prompts to reach out to ECEC educators to discuss any changes in their child’s diet or feeding.
- Access to a digital toolbox which houses a codesigned evidence-based collection of infant feeding and responsive feeding resources called the Lumpy Road to Solids, situated on the The Grow & Go Toolbox website funded by the Australian Government. Some SMS messages and e-newsletters will include links that direct caregivers to this webpage and/or specific resources.
- Access to the infant feeding and responsive feeding webinars developed to support ECECs with implementation (see above for more detail).

#### Control: ECEC services and primary caregivers

ECEC services allocated to the control group will receive usual care, which includes the Munch and Move Programme. This programme is funded by the NSW government where health promotion support is provided to help ECEC services improve their nutrition and physical activity environments. While the programme includes support to improve breastfeeding environments, it does not target educator infant feeding and responsive feeding practices targeted in this study.^54^ Primary caregivers will have access to usual child and family health websites and government resources^53^ including webpages like the Raising Children Network and other freely available parenting resources; however, this will need to be actively sought out by parents. Parents will also be able to access child and family health services for routine developmental checks. To prevent contamination, the web-based online resources and training for ECECs will be accessible to intervention services only for the duration of the trial. The research team will manage the delivery of the caregiver intervention. Similarly, caregiver intervention resources are not publicly accessible and require a private link to ensure only intervention caregivers are accessing information.

### Outcome measures

#### zBMI

The primary outcome is child zBMI (measured twice at baseline and at 18 months follow-up (postintervention)). Measurements will be undertaken in an allocated area of the ECEC service by a research team member blinded to group allocation with appropriate child protection clearance and training in anthropometric measurement of infants.^55^ Research assistants will attend a 3-hour face-to-face training session facilitated by one researcher (MLu) to ensure familiarity with the data collection protocol. Children will be in light clothing, without shoes/jackets and with nappies on. Measurements will be completed following a standardised protocol for measuring infants.^56^ Weight will be measured with the child in a lying or seated position using a calibrated digital scale (Seca baby measuring scale, Model SE374) and recorded to the nearest 10 grams. Length will be measured with the child in a supine position on a level floor (with a Wedderburn Baby Measuring Mat Model WMHM110M) and recorded to the nearest 0.5 cm on a hard, flat surface. At follow-up, standing height will be measured barefooted using a mobile stadiometer (Charder HM 200P, in cm, to 10 mm increments). Two weight and length/height measurements will be taken at each time point. Where the measurements differ, a third measurement will be taken. The most common repeated measure will be used, or where all three measures differ, the average measure will be used.

#### Secondary outcomes

The following will be assessed at baseline and 18-month follow-up.

Primary caregiver outcomes

- Duration of any breast milk. Caregivers will be asked about current and past breastfeeding to determine duration of breastfeeding (completed months) and exclusive breastfeeding to 6 months if applicable.
- Child variety and frequency of fruit and vegetable intake (daily) Intake (at home) will be assessed using items from a Food Frequency Questionnaire^57^ and the Children’s Dietary Questionnaire, which has been validated in children aged 2–5 years old.^58^ The variety, frequency and quantity of fruit and vegetables consumed by the child will be captured, and an overall score for variety and frequency will be calculated.
- Child frequency of consuming discretionary food and sugar-sweetened beverage intake (daily, weekly or monthly). Intake (at home) will be measured using eight items from the validated Food Frequency Questionnaire.^57^ This will capture the frequency of discretionary food consumption, specifically processed meats, biscuits, fried foods and sugar-sweetened beverages.
- Caregiver adherence to infant feeding recommendations and responsive feeding practices. Questions on infant feeding practices, developed by the research team, will assess the timing of introduction to solid foods and drinking from a cup, and general feeding style. Responsive feeding practices will be evaluated using an adapted version of the validated Feeding Practices and Structure Questionnaire.^59^ We will assess how often caregivers appropriately respond to hunger and satiety cues, engage in persuasive feeding or use food as a reward.^59^ Items will be measured on a 5-point Likert scale from never to always. The responses to each item will be summed to provide a score for each construct.

#### ECEC service outcomes

- Comprehensive nutrition and breastfeeding policies. ECEC services will be asked to provide any infant and toddler nutrition/healthy eating or breastfeeding policies they adhere to. Policies will be reviewed and assessed for content from the NHMRC infant feeding guidelines,^14^ a statement of support for breastfeeding provided at first contact with families, documented feeding plans, responsive feeding practices and educator training.
- Educator infant and responsive feeding practices. Five items based on the adapted Environment and Policy Assessment and Observation (EPAO) tool^60^ exploring the frequency of a range of infant feeding practices for children aged 2 years and under will be asked to one nominated educator within the infant room. These practices are related to bottle feeding, using bottles or food to soothe children, encouraging children over 6 months to drink from a cup and use age-appropriate cutlery, as well as progressing food textures appropriately. They will be rated on a 7-point Likert scale from never to always. The educator will also be asked 14 questions exploring responsive feeding: (1) education/modelling (three items), (2) using food as a reward (four items), (3) mealtime environment (three items) and (4) use of coercive practices (four items). The items are rated on a 5-point Likert scale from never to always and are based on the Adapted About Feeding Children Strategies and Beliefs survey.^61^ The responses to each item will be summed to provide an overall score, as well as a score for each construct. At follow-up only, we will also undertake researcher observations during a main mealtime, where most children are present (typically lunch) to assess delivery of the targeted feeding practices using an adapted version of the EPAO previously employed by the research team.^62^

#### Intervention cost (cost-effectiveness outcome)

Costs associated with initial intervention development and intervention delivery/implementation will be recorded by the research team and collected via REDCap throughout the study period. Development costs related to the web-based platform, suite of text messages, training modules and resources will be documented by the research team via REDCap. Direct costs for intervention delivery will include staff time (ECEC staff, HPOs, administrative support), expert fees for webinar delivery, physical resource provision, website maintenance, text-message and e-newsletter platform expenses, and training and ongoing support to educators. Both will be documented by the research team via REDCap. ECEC educators will report on staff time and resources used on infant nutrition via the survey at baseline and follow-up. For primary caregivers, costs will be measured by estimating the time spent engaging with the intervention materials. This will be captured through website software analytics and by estimating time required to complete education modules if accessed. Similarly, primary caregivers will be asked to estimate how much money and time they spend feeding their child at baseline and follow-up to allow for comparisons between groups.

#### Economic evaluation

To determine cost-effectiveness, the total cost of the intervention will be presented disaggregated for varied stakeholders including: (1) the health service alone, (2) health and ECEC services and (3) health+ECEC services+primary caregivers. The total costs will also be estimated with and without the cost of intervention development (assuming the intervention is in steady state). The incremental cost of the intervention (cost of intervention–cost of usual care) will be calculated. A cost–consequence analysis will be conducted, presenting incremental costs of the intervention along with the differences in all outcome measures. Cost-effectiveness analyses will present the additional cost per 0.1 zBMI reduction. The long-term benefit of improvements in child zBMI will be modelled to longer term outcomes (health adjusted life years gained) using a previously developed and validated proportional multistate, multiple cohort lifetable model.^63^

#### Adverse effects

In services where food is provided, ECEC managers will be asked about the percentage of food wastage for children aged 2 years and under, and the number of complaints or concerns raised by caregivers and staff around infant feeding and nutrition at baseline and follow-up. Questions will be developed by the research team.

### Process evaluation measures

The following evaluation measures will be reported on at follow-up.

- Engagement: We will track access and use of the web programme by ECEC services using website analytics (eg, number of webinar views). The completion status of the action plan for each service will be monitored. We will also record the number of primary and secondary caregivers interacting with monthly e-newsletters (eg, open/click rate) and accessing embedded resource links (ie, Lumpy Road to Solids website and resources). Finally, we will report the number of text messages successfully delivered.
- Acceptability: Intervention ECEC managers and educators will be asked to report on the acceptability of the intervention using four items based on the validated Acceptability of Intervention Measure tool.^64^ Caregivers will be asked to assess the acceptability of the intervention components using measures developed by the research team.
- Feasibility: Feasibility will be assessed with ECEC services via a survey using four items (three questions on a Likert scale and one open-ended item) adapted from the Feasibility of Intervention measure.^64^
- Fidelity: Fidelity of HPO implementation strategies within ECEC services will be measured using monitoring instruments within REDCap.

### Other outcome data

#### Demographic data

##### ECEC characteristics

- At baseline, we will collect days and hours of operation, service size (number of children), number of educators (including those in the infant room), number of children in infant room, number of culturally and linguistically diverse child enrolments, whether they provide all food on site, and whether they are receiving any other active interventions.

##### Caregiver and child demographic data

- Sociodemographic variables collected at baseline will include primary caregiver’s year of birth, relationship to child, cultural background, employment type, timing of return to work following birth (where applicable), marital status, experience parenting (first time), education level, self-reported height and weight, perception of income stress, current pregnancy status, breastfeeding status, and whether they are receiving ongoing support around infant feeding. Primary caregivers will also be asked about their child’s height and weight, living arrangements and enrolment at other ECEC services.

At baseline and follow-up, the following variables will also be assessed, as they represent likely mechanisms through which the intervention may exert its effects, as outlined in figure 2.

### Primary caregiver–ECEC provider relationship

We will assess caregiver–educator partnerships using an adapted version of the Parent/Teacher Relationship Quality Scale.^65^ Primary caregivers (8 items) and educators (11 items) will each complete a separate version of this scale to assess trust in communication regarding children’s nutrition. Each item is rated on a 5-point Likert scale from strongly disagree to strongly agree. The responses to each item will be summed to provide an overall score.

### ECEC educator and caregiver self-efficacy

Educators will be asked to rate their confidence with delivering various responsive feeding practices. Responses will be rated on a 5-point Likert scale from ‘not at all confident’ to ‘extremely confident’. Similarly, primary caregivers will complete self-efficacy questions assessing their confidence in relation to child feeding practices. Questions are based on the Maternal Self-Efficacy scale.^66^

### Communication between ECECs and caregivers

ECEC managers will be asked about the following types of communication with caregivers using questions previously used by the research team. Questions will address:

- The type of breastfeeding and infant feeding information provided to newly enrolled primary caregivers of infants.
- Availability of information regarding breastfeeding and infant feeding on the website.
- Education provided to families in the last 12 months regarding infant nutrition through workshops, webinars or meetings.
- Whether infant feeding information from a recognised health authority was shared with families in the last 12 months.
- Whether ECECs display posters, stickers or signage promoting healthy infant feeding and breastfeeding.

ECEC managers^1^ will also be asked if educators have access to resources to facilitate nutrition conversations with primary caregivers as needed. Educators will also be asked about the frequency with which they provide feeding and nutrition information to primary caregivers regarding their children (eg, types and amounts of foods consumed, new foods they have tried, progression of self-feeding skills) at baseline and follow-up.

### Data collection and trial management

Data will be handled in accordance with ethical guidance and accessible only to named researchers on the ethics application. All electronic data will be stored on password-protected files on secure servers at Deakin University. Intervention data (eg, ECEC recruitment and randomisation schedules) will also be securely stored at HNE, as they are responsible for intervention delivery in the region. Primary caregivers’ mobile numbers will be shared with the HNE Population Health HB4HNE Kids team to avoid duplicate messaging. Paper forms will be scanned, electronically stored on secure servers and then destroyed.

The advisory group, consisting of all chief and associate investigators from all partner organisations listed on the ethics application, will meet quarterly to discuss trial conduct and audit trial progress.

### Data and safety monitoring

We do not anticipate any serious adverse events as a unique consequence of participation in this study. In the unlikely event this occurs, it will be reported to the Human Research Ethics Committee. A Data Monitoring Committee is not required for this study, as the intervention is non-clinical, poses minimal risk and does not involve the administration of a drug or investigational product. No interim analyses are planned.

### Sample size calculation

The sample size calculation was based on the primary outcome, zBMI. Based on our previous study,^55^ the SD is approximately 1.0 and mean zBMI is approximately 0.41. To detect a clinically meaningful change in population zBMI of 0.26 (69) with 80% power (two-sided α=0.05, intracluster correlation coefficient=0.02), we will need to recruit 540 caregiver–child pairs (270 per group) from 60 ECEC services. As an intention-to-treat analysis will be undertaken as the main analysis (participants analysed according to allocation group), we will not account for attrition.

### Statistical analysis

The primary analyses will be undertaken using an intention-to-treat approach, using multiple imputation to account for missing data. For the primary outcome (continuous zBMI), between-group differences at follow-up will be assessed using hierarchical linear regression models. We will also dichotomise the data to assess overweight/obese (zBMI using WHO cut-offs)^67^ and compare this using a logistic regression. All models will be adjusted for ECEC service-level clustering through a random effect and controlled for baseline measures and ECEC service SES and any other identified baseline imbalances. To assess differences between groups for secondary outcomes, linear regression analyses will be conducted (for continuous outcomes). Models will be adjusted for ECEC service-level clustering and any stratified variables through a random effect and controlled for baseline measures and ECEC service SES. Similar analysis will be undertaken for child-level secondary outcomes. All model inference will be conducted within the Bayesian framework. Sceptical, mean-centred normal prior distributions will be specified for the treatment effect and other fixed-effect regression parameters. A half-Student-t distribution will be used to model the SDs of random effects and residuals. Posterior draws will be obtained using the No-U-Turn Sampler, as implemented in the brms package for R.^68^ The probability of a favourable treatment effect (ie, the posterior probability that the treatment effect is greater than zero) will be reported, along with 95% credible intervals for the treatment effect parameters.

## Ethics and dissemination

### Human research ethics approval

The trial was prospectively registered with the Australian New Zealand Clinical Trials Registry (ACTRN12624000576527). Ethics approval has been provided by Hunter New England (HNE) Human Research Ethics Committee (HREC) (2023/ETH01158), Deakin University (2024-202) and University of Newcastle HREC (R-2024-0039). Any changes to the approved protocol will be documented and submitted as formal amendments to the HREC.

### Privacy and confidentiality

Only research staff directly involved in the study will have access to identified participant data. Data will be deidentified using study ID numbers. Personal identifiable information (names, contact details) will be kept separately from outcome data and will be accessible only by research personnel.

### Patient and public involvement

We co-designed the Tiny Bites programme with the following interest-users (caregivers of young children attending ECEC services, health promotion teams, ECEC managers and ECEC educators), who were also consulted prior to development of the funding application. Most of the intervention materials were pilot tested with consumers prior to delivery and we drew on co-design workshops with ECEC providers, health care professionals and cargivers undertaken as part of the Grow and Go design (led by HT and CD). The health service delivery (HNELHD) and state health department who funds implementation support for ECEC services (NSW Centre for Population Health) are partners on the funding application and authors on this protocol.

### Dissemination plan

The results of this trial will be published in a peer-reviewed journal and presented at academic conferences/seminars. Results will be presented in a variety of formats including, but not limited to, conference abstracts, posters or presentations, seminars, journal articles and internet postings.

## Supplementary material

10.1136/bmjopen-2025-106436 online supplemental file 1

10.1136/bmjopen-2025-106436 online supplemental file 2

10.1136/bmjopen-2025-106436 online supplemental file 3
