# Supplementary material for: Tiny Bites, a digital health intervention delivered in early childhood education and care centres to support educators and caregivers to prevent childhood obesity: study protocol for a cluster randomised controlled trial
Source: BMJ Open. 2025 Nov 23;15(11):e106436. doi: 10.1136/bmjopen-2025-106436 (PMC12645655; doi:10.1136/bmjopen-2025-106436)
Supplement: online supplemental file 1 [file bmjopen-15-11-s001.pdf]

Hunter New England Population Health
Phone: (02) 4924 6477 Fax: (02) 4924 6490
Locked Bag 10, Wallsend NSW 2287
Email: HNELHD-PHEnquiries@health.nsw.gov.au
www.hnehealth.nsw.gov.au

The Tiny Bites program – a randomised controlled trial assessing the impact of an intervention to support parents and childcare services with healthier diets in children aged <2 years

SERVICE CONSENT FORM
Version 3, 28/03/2024

I have been made aware of the procedures involved in participating in the abovementioned study, including:

- services will be randomly allocated to one of two groups (either to receive the Tiny Bites program or usual practice group);
- data collection will occur at baseline and 18 months follow-up with the nominated supervisor and an educator in the nursery/baby room;
- any risks or benefits as far as they are currently known by the researchers.

I consent to the members of the research team visiting my service as detailed in the Participant Information Statement.

I understand that my service can withdraw at any time without providing a reason. This will in no way disadvantage my service, nor will it affect my service's relationship with the *Good for Kids. Good for Life* program.

I understand that my and my services personal information will remain confidential to the researchers.

I have had the opportunity to have questions answered to my satisfaction.

By signing below, I am indicating my consent for my service to participate in this study, as it has been described to me in the Participant Information Statement, a copy of which I have retained.

Name of service:

____

Nominated Supervisor's name:

____

Nominated Supervisor's signature:

____

Date:

Best day/time to contact you:

Nominated Supervisor email address:

Please nominate a Room Leader or Educator in the nursery/baby room to contact around Tiny Bites.

Name of staff member:

Best day/time to contact staff member:

Staff member email address:

Please complete and return to:
< insert appropriate contact >
