# Supplementary material for: Tiny Bites, a digital health intervention delivered in early childhood education and care centres to support educators and caregivers to prevent childhood obesity: study protocol for a cluster randomised controlled trial
Source: BMJ Open. 2025 Nov 23;15(11):e106436. doi: 10.1136/bmjopen-2025-106436 (PMC12645655; doi:10.1136/bmjopen-2025-106436)
Supplement: online supplemental file 2 [file bmjopen-15-11-s002.pdf]

Hunter New England Population Health
Phone: (02) 4924 6477 Fax: (02) 4924 6490
Locked Bag 10, Wallsend NSW 2287.
Email: HNELHD-PHEnquiries@health.nsw.gov.au
www.hnehealth.nsw.gov.au

The Tiny Bites program – a randomised controlled trial assessing the impact of an intervention to support parents and childcare services with healthier diets in children aged <2 years

PRIMARY CAREGIVER CONSENT FORM
Version 2, 24/04/2024

I have been made aware of the procedures involved in participating in the abovementioned study, including:

- the randomisation of services, and therefore myself and my child will be allocated to one of either two groups (Tiny Bites Program or usual practice group);
- data collection at baseline and 18 months follow-up; if you have indicated you are already receiving the Healthy Beginning for HNE Kids text message program (or are unsure), sharing of mobile phone information to their team to prevent duplication; and
- any risks or benefits as far as they are currently known by the researchers.

I understand that I can withdraw at any time without providing a reason and this will not disadvantage me or my child, nor will it affect my child's relationship with their early childhood education and care service.
I understand that my and my child's personal information will remain confidential to the researchers.

I have had the opportunity to have questions answered to my satisfaction.

I confirm I use a mobile phone with internet capabilities.

By signing below, I am indicating my consent to participate in this study, as it has been described to me in the Participant Information Statement, a copy of which I have retained.

Primary Caregiver Name: ______________________________

Primary Caregiver Signature: ______________________________ **Date:** ______________

____

Mobile phone no: ***Preferred:*** ______________ Alternate: ______________

What are the best days and times to contact you via phone? ______________________________

Email address: ***Preferred:*** ______________________________

Alternate: ______________________________

Your child's name: First name: ______________________________

Last name: ______________________________

Child's date of Birth (DD/MM/YYYY) ______________________________

Child's sex (*please* ☑) ☐ Male ☐ Female

Home postcode (where your child spends the most time): ______________________________

Childcare service name: ______________________

Number of days your child attends this childcare service ______________________

Days your child attends this childcare service (*please* ☑ all that apply) ☐ Mon ☐ Tues ☐ Weds ☐ Thurs ☐ Fri

Does your child have special dietary requirements? *If yes, please provide further information* ______________________

Are you currently receiving the HNE for Healthy Beginnings text messages? ☐ Yes ☐ No ☐ Unsure

If you would also like to elect an additional caregiver of your child (if applicable) for us to contact with information, please provide their details below. If you are allocated to receive the intervention, we will also send them e-newsletters about infant feeding.

Additional Caregiver Contact Name: ______________________

Relationship to child: ______________________

Mobile phone no: ***Preferred:*** __________ Alternate: __________

What are the best days and times to contact the additionalCaregiver? ______________________

Email address: ***Preferred:*** ______________________

Alternate: ______________________

Please complete and return to:
< insert appropriate contact >
