# Supplementary material for: Tiny Bites, a digital health intervention delivered in early childhood education and care centres to support educators and caregivers to prevent childhood obesity: study protocol for a cluster randomised controlled trial
Source: BMJ Open. 2025 Nov 23;15(11):e106436. doi: 10.1136/bmjopen-2025-106436 (PMC12645655; doi:10.1136/bmjopen-2025-106436)
Supplement: online supplemental file 3 [file bmjopen-15-11-s003.docx]

| **Caregiver level determinants to improve infant feeding practices and diet** | **Modality** | **COM-B domain** | **Intervention function (from Behaviour Change Wheel)** | **BCTs used** |
| --- | --- | --- | --- | --- |
| 1. Caregiver knowledge and self-efficacy around feeding | Newsletter | Capability- Psychological  Motivation-Reflective  Opportunity-Social  Opportunity-Physical | Education  Persuasion  Training  Modelling | 3.1 Social support (unspecified)  4.1 Instruction on how to perform the behaviour  4.2 Information about Antecedents  4.3 Re-attribution  5.1 Information about health consequences  7.4 Reduce access to the reward  7.5 Remove aversive stimulus  7.8 Associative learning  8.1 Behavioural practice/rehearsal  8.2 Behavioural substitution  11.2 Reduce negative emotions  12.1 Restructuring the physical environment  15.2 Mental rehearsal of successful performance |
|  | Text messages | Same as above | Education  Persuasion  Training | 3.2 Social support (emotional)  4.1 Instruction on how to perform a behaviour  4.2 Information about antecedents  4.3 Re-attribution  7.1 Prompts/cues  7.5 Remove aversive stimulus  8.1 Behavioural practice/rehearsal  8.2 Behavioural substitution  12.1 Restructuring the physical environment  13.1 Identification of self as role model  15.2 Mental rehearsal of successful performance  15.3 Focus on past success  15.4 Self-talk |
|  | Website | Same as above | Education  Persuasion  Training  Environmental restructuring  Modelling | 3.1 Social support (unspecified)  3.2 Social support (emotional)  4.1 Instruction on how to perform a behaviour  4.2 Information about antecedents  4.3 Re-attribution  5.1 Information about health consequences  6.1 Demonstration of the behaviour  7.4 Remove access to the reward  7.5 Remove aversive stimulus  7.8 Associative learning  8.1 Behavioural practice/rehearsal  8.2 Behaviour substitution  11.2 Reduce negative emotions  12.1 Restructuring the physical environment  13.1 Identification of self as role model  15.2 Mental rehearsal of successful performance  15.3 Focus on past success  15.4 Self-talk |
| 2. Conflicting info and advice | Caregiver resources and the Lumpy Road  Newsletter  ECEC communication to caregivers | Capability- Psychological  Opportunity-Social | Education  Persuasion  Environmental restructuring  Main strategy we used here is distilling info from evidence (removing conflicting advice) and presenting it in the resources  Also providing info to:  Caregivers (from us)  ECEC  Caregivers (via ECEC)  That are consistent and aligned, to minimise confusion | 8.2 Behaviour substitution  9.1 credible source  11.3 conserving mental resources  12.2 restructuring the social environment |
| 3. Caregiver concerns for child health (if not eating) | Resources  Newsletter  Text | Capability- Psychological  Motivation-Reflective  Opportunity-Social | Education  Persuasion  Training  Modelling | 3.1 Social support (unspecified)  4.1 Instruction on how to perform a behaviour  4.2 Information about antecedents  4.3 Re-attribution  5.1 Information about health consequences  7.5 Remove aversive stimulus  8.2 Behaviour substitution  13.2 framing/ reframing |
| 4. **Lack of access to reliable and timely info** | Resources & Lumpy Road  Text | Capability- Psychological  Opportunity-Social  Opportunity-Physical | Education  Environmental restructuring  Enablement | 3.1 Social support  7.1 Prompts/cues  8.2 Behaviour substitution  9.1 credible sources  12.1 Restructuring the physical environment |
| 5. Perception of **lack of consequences** regarding different feeding styles | Resources and Lumpy Road  Newsletters  Text | Capability- Psychological  Opportunity-Social  Opportunity-Physical | Education  Persuasion | 4.3 Re-attribution  5.1 Information about health consequences  5.3 Information about social and environmental consequences  13.2 framing/ reframing |
| 6. **Unsure** about how ECECs can support families (to ensure information is delivered by trusted source, **ECEC as the trusted source** of nutrition information). | Newsletters  Texts  ECEC communication to caregivers | Capability- Psychological  Opportunity-Social  Motivation-Reflective | Education  Persuasion  Environmental restructuring | 4.1 Instruction on how to perform a behaviour  6.1 Demonstration of the behaviour  7.1 Prompts/Cues  12.2 Restructuring the social environment |
| 7. Potential **conflict** in feeding behaviours **in care and home**. | Newsletters  Chat topics to ECECs | Capability- Psychological  Opportunity-Social  Opportunity-  Physical  Motivation-Reflective | Education  Persuasion  Training | 4.1 Instruction on how to perform a behaviour  7.1 Prompts/ cues  8.2 behaviour substitution  9.1 credible sources  10.6 non-specific incentive  12.2 restructuring the social environment |
| ECEC reported determinants to implementation | | | | |
| Barriers and enablers | Implementation strategies | COM-B domain | Intervention Functions | BCT behaviours |
| Supervisor support with implementing the program and supporting staff to attend training | Educational outreach | Capability- Psychological | Education Enablement | 5.1 Information about health consequences  5.3 Information about social and environmental consequences |
| Service viewing their role as a more passive role | Training (online webinar) | Motivation-Reflective (beliefs about consequences, beliefs about capabilities) | Education  Persuasion | 5.1 Information about health consequences  5.3 Information about social and environmental consequences  9.1 credible sources |
| Lack of comprehensive policy and procedures to support infant feeding | Develop and distribute educational materials | Capability-Psychological (Knowledge, memory, attention and decision processes)  Reflective Motivation (beliefs about capabilities) | Education  Enablement | 4.1 Instruction on how to perform a behaviour  5.1 Information about health consequences  7.1 Prompts/ cues |
| Educators lack of knowledge, skills, and confidence. | Training  Educational outreach  Develop and distribute educational materials  Provision of feedback | Physical- Capacity (knowledge, memory, attention and decision processes)  Opportunity- social (social influences)  Motivation-reflective (Goals, beliefs about capabilities) | Persuasion  Enablement  Education  Modelling | 7.1 Prompts/ cues  Review behaviour goals  4.1 Instruction on how to perform a behaviour  6.1 Demonstration of the behaviour  2.2 Feedback on behaviour |
| Educators’ attitude, beliefs and perceived priority regarding implementing recommendations. | Develop formal implementation blueprint  Facilitation  Provision of feedback  Identify and prepare champions | Motivation-Reflective (Goals)  Behavioural cueing (other MOA)  Capability- psychological  Motivation -reflective  Opportunity- reflective (social influences) | Persuasion  Enablement  Education  Modelling | 1.5 Action Planning  7.1 Prompts/ cues  Review behaviour goals  4.1 Instruction on how to perform a behaviour  6.1 Demonstration of the behaviour  15.1 Verbal persuasion about capability |
| Caregivers’ existing beliefs regarding infant feeding (potential conflict with ECECs) | Develop and distribute education materials | Capability-Psychological (Knowledge, memory, attention and decision processes)  Reflective Motivation (beliefs about capabilities) | Education  Enablement | 4.1 Instruction on how to perform a behaviour  5.1 Information about health consequences  7.1 Prompts/ cues |
| Lack of processes to support timely communication with caregivers | Develop and distribute education materials | Capability-Psychological (Knowledge, memory, attention and decision processes)  Reflective Motivation (beliefs about capabilities) | Enablement | 7.1 Prompts/ cues |

- BCT; behaviour change technique, COM-B; capability, opportunity, motivation, behaviour, ECEC; early childhood education and care, MOA; mechanisms of action
