# Supplementary material for: Tiny Bites, a digital health intervention delivered in early childhood education and care centres to support educators and caregivers to prevent childhood obesity: study protocol for a cluster randomised controlled trial
Source: BMJ Open. 2025 Nov 23;15(11):e106436. doi: 10.1136/bmjopen-2025-106436 (PMC12645655; doi:10.1136/bmjopen-2025-106436)
Supplement: Supplementary file 4 [file bmjopen-2025-106436.reviewer_comments.pdf]

PEER REVIEW HISTORY

BMJ Open publishes all reviews undertaken for accepted manuscripts. Reviewers are asked to complete a checklist review form and are provided with free text boxes to elaborate on their assessment. These free text comments are reproduced below.

ARTICLE DETAILS

Title (Provisional)

Study protocol for a cluster randomised controlled trial of a digital health intervention (Tiny Bites) delivered in early childhood education and care centres to support educators and caregivers to prevent childhood obesity

Authors

Yoong, Sze Lin; Lum, Melanie; Leung, Gloria KW; Pearson, Nicole; Dix, Clare; Wiggers, John; Delaney, Tessa; Romiti, Maria; Lamont, Hannah; Stanley, Sonya; Lim, Michelle; Mastersson, Nadia; Truby , Helen; Moumin, Najma A; Wolfenden, Luke; Ananthapavan, Jaithri; Grady, Alice; Rychetnik, Lucie; Oldmeadow, Chris; Sutherland, Rachel

---

VERSION 1 - REVIEW

**Reviewer**	**1**
**Name**	**Birnie, Erwin**
**Affiliation**	**Franciscus Gasthuis en Vlietland, Statistics and Education**
**Date**	**17-Sep-2025**
**COI**	**None**

This is an interesting and potentially relevant cluster-RCT on the efficacy, effectiveness and implementational aspects of the digital Tiny Bites intervention that is aimed to support the implementation of “good” infant feeding recommendations in the short run, to prevent obesity in the longer run.

Strengths of the protocol: strong study rationale, well considered, well written, randomized design (Tiny Bites implementation vs. usual care), taking the implementational aspects and socio-economic and socio-cultural context into account (skills, access to information, the concept of responsive feeding, rural environment, parental return to work, socially disadvantaged regions or groups). Trial registration and ethics approval have already been obtained.

Potential weaknesses are the data collection that is quite ambitious and the use of questionnaires that make part of the outcomes vulnerable to non-response.

Being aware of the "Notes From The BMJO Editors For Study Protocol", the following considerations can be made:

1. Effectiveness (and cost-effectiveness) of the Tiny Bites intervention appears to depend on the compliance to the feeding recommendations, and determinants explaining compliance, at the ECEC as well as primary caregivers' levels. This is largely done using questionnaires, which make the study results vulnerable to (selective?) non-response.

2.What is currently considered usual care?

3.P13, Table 1. The box(es) for the Assessments has (have) not been ticked. Reading the protocol, the Assessments probably are made in the follow-up period (BMI z-scores), but probably also during the study?

4.P14, Randomisation. The study and control ECEC services will not be matched?

5. P26, Sample Size Calculation. It would be useful to have references of previous studies that are in support of the assumptions underlying the sample size calculation.

6. SPIRIT item 5b. This item is ticked N/A, but all studies have a sponsor. Given the leading role of FY, is this perhaps the Deakin University or University of Newcastle?

VERSION 1 - AUTHOR RESPONSE

Reviewer 1		
	COMMENTS TO THE AUTHOR<br>This is an interesting and potentially relevant cluster-RCT on the efficacy, effectiveness and implementational aspects of the digital Tiny Bites intervention that is aimed to support the implementation of "good" infant feeding recommendations in the short run, to prevent obesity in the longer run.<br>Strengths of the protocol: strong study rationale, well considered, well written, randomized design (Tiny Bites implementation vs. usual care), taking the implementational aspects and socio-economic and socio-cultural context into account (skills, access to information, the concept of responsive feeding, rural environment, parental return to work, socially disadvantaged regions or groups). Trial registration and ethics approval have already been obtained.<br>Potential weaknesses are the data collection that is quite ambitious and	Thank you for the helpful reviewer comments

	the use of questionnaires that make part of the outcomes vulnerable to non-response	
7	Effectiveness (and cost-effectiveness) of the Tiny Bites intervention appears to depend on the compliance to the feeding recommendations, and determinants explaining compliance, at the ECEC as well as primary caregivers' levels. This is largely done using questionnaires, which make the study results vulnerable to (selective?) non-response.	The primary outcome will be obtained via measured weight and height while the secondary outcomes are primarily self-reported. Where possible we have used questionnaires with established validity. However, we agree that this is a limitation of the study and have acknowledged this in the strengths and limitation section (see page 4).<br><br>The use of self-report assessment for secondary outcomes may be subject to reporting and attrition bias.
8	What is currently considered usual care?	We have provided some additional information about usual care for ECECs and primary caregivers on pages 18 and 19 of the manuscript.<br><br>This program is funded by the NSW government where health promotion support is provided to help ECEC services improve their nutrition and physical activity environments. While the program includes support to improve breastfeeding environments, it does not target educator infant feeding and responsive feeding practices targeted in this study. [54] Primary caregivers will have access to usual child and family health websites and government resources [53] including webpages like the Raising Children Network and other freely available parenting resources however this will need to be actively sought out by parents. Parents will also be able to access child and family health services for routine developmental checks.
9	P13, Table 1. The box(es) for the Assessments has (have) not been ticked. Reading the protocol, the Assessments probably are made in the follow-up period (BMI z-scores), but probably also during the study?	BMI z-scores (child weight and height)) are only assessed at baseline and follow up by researchers at the ECEC services, and not during the study<br>We have amended the manuscript so this is clearer (see page 19).

		The primary outcome is child BMI (z-score, measured twice at baseline and at 18-months follow-up (post intervention))
10	P14, Randomisation. The study and control ECEC services will not be matched?	For this trial, we have not matched the intervention and control arm as we have a relatively large number of clusters. Where an imbalance is identified, we will adjust for this through co-variate adjustments in the mixed-effects models (see page 28)<br><br>“All models will be adjusted for ECEC service-level clustering through a random effect and controlled for baseline measures and ECEC service socioeconomic status and any other identified baseline imbalances.”
11	5. P26, Sample Size Calculation. It would be useful to have references of previous studies that are in support of the assumptions underlying the sample size calculation.	Thank you for this comment – we have added the references to this section (see page 27, ref 55 and 69).
12	SPIRIT item 5b. This item is ticked N/A, but all studies have a sponsor. Given the leading role of FY, is this perhaps the Deakin University or University of Newcastle?-	Apologies for this error – we have now amended this to reflect that Deakin University as the sponsor on page 4 with contact and the role of the sponsors included in the funding contributions and updated the SPIRIT checklist.<br><br>“The sponsor (Deakin University) and funders will have no role in the design, data collection, analysis, interpretation of results, or decision to publish the findings.”
